# L2 Enjoyment of English as a Foreign Language Students: Does Teacher Verbal and Non-verbal Immediacy Matter?

**DOI:** 10.3389/fpsyg.2022.897698

**Published:** 2022-06-20

**Authors:** Hongyu Guo, Wurong Gao, Yumin Shen

**Affiliations:** ^1^School of Foreign Languages, Zhejiang Gongshang University, Hangzhou, China; ^2^Graduate School of Education, University of Perpetual Help System DALTA, Las Piñas, Philippines

**Keywords:** foreign language enjoyment, positive psychology, teacher immediacy, teacher-learner rapport, teacher-learner relationship

## Abstract

This review explored the investigations on the role of teacher immediacy in English as a Foreign Language (EFL) learners’ foreign language enjoyment. Earlier investigations have proved that teacher immediacy, such as posture, body language, vocal variety, gestures, and smile, can significantly affect learners’ positive emotions like foreign language enjoyment. It means that teachers should try both to control the feelings of their learners and manage their feelings to enhance enjoyment among learners. Moreover, studies have shown that teacher immediacy is significantly related to learner motivation which is a basis for increasing foreign language enjoyment among learners. However, specific strategies can be employed to increase learner motivation, which in return increases learner foreign language enjoyment. Furthermore, the study presented the implications and future directions of this line of research for different people, such as EFL teachers, teacher educators, and foreign language scholars. The ideas can improve their awareness of teacher-student relationships, in particular, teacher immediacy and its role in learners’ foreign language enjoyment.

## Introduction

Interaction is regarded as one of the most critical issues in educational contexts. Malik (2012) states that instruction is an interaction-based performance. To increase the effectiveness of this interaction, it is important for teachers to have language proficiency, the capability of designing approaches and methods, the ability to assess and evaluate learners, and the aptitude to use numerous instruction aids. Moreover, they should build their relationship with learners by considering their negative and positive affectivities (Liu, 2016). The formation of educational context and finding proper methods to entice learners to make teaching a demanding job (Inan-Kaya and Rubie-Davies, 2021). Liando (2010) stated that teacher immediacy is considered one of the teaching variables that is significant in EFL contexts. Andersen (1979) incorporated immediacy in the educational context and, defined teacher immediacy as “a concept which describes communication behaviors that reduce the perceived distance between the students and teachers, in instructional communication” (p. 20). On the other hand, foreign language enjoyment, as the positive psychology construct, has drawn the attention of positive psychologists, since they try to enhance learning outcomes and foster learning contexts (Wang et al., 2021). Reeve (2005) stated that foreign language enjoyment refers to the feeling of “desirable outcomes related to personal success and interpersonal relatedness” (p. 316). Concepts such as enjoyment, psychological engagement, motivation, wellbeing, pride, hope, and teachers’ teaching variables, and pedagogical love were considered to be significant variables in improving the performances of the teachers and learners in the literature (Frymier et al., 2019). However, there have been few previous investigations of teachers’ verbal and non-verbal immediacy and their effect on learner enjoyment which make it necessary for investigators to do research in this field. Having an awareness of teachers’ verbal and non-verbal immediacy, and their roles in learner enjoyment can endorse and expand the positive psychology constructs. Moreover, by knowing about these variables, school managers would be able to make their teachers enthusiastic and improve their positive emotions to enhance learner enjoyment. This review tries to investigate the related literature about the role of teachers’ verbal and non-verbal immediacy in learner foreign language enjoyment.

## Review of Literature

### Teacher’s Verbal and Non-verbal Immediacy

The teacher-learner relationship is one of the most important features of instruction. One of the components of teacher-learner interaction is teacher immediacy. According to Sheybani (2019), immediacy refers to “degree of perceived physical or psychological closeness between people” (p. 68). Wen and Clement (2003) emphasized that teacher immediacy can be verbal or non-verbal, such as verbally using humor and personal anecdotes or using body language like head nods or smiles. Velez and Cano (2012) defined verbal immediacy as the formal differences in teachers’ utterances, based on which learners decide to like or dislike the utterance of a teacher. Rocca (2007) also pointed out that verbal immediacy refers “to communication behaviors such as calling students by names, asking for students’ feedback about the lessons, referring to the class as “we” and “our,” and engaging in conversations with students before and after class” (p. 43). Verbal immediacy also comprises implications for conveying compassion, clarity, sympathy, reward, admiration, funniness, individual knowledge, and educators’ readiness to include learners in interaction (Pladevall-Ballester, 2015).

On the other hand, Peng (2020) defined non-verbal immediacy as any kind of approach that instructors may implement to decrease their mental or physical detachments with their learners. Witt and Kerssen-Griep (2011) also mentioned that non-verbal immediacy can be signified by teachers’ eye-contact, gestures, posture, body language, laughing, and employing vocal variety, which affects teacher-learner rapport. Andersen (1979) stated that non-verbal immediacy may deliver emotional states of warmness, intimacy, and belonging. Peng et al. (2016), in their study about a non-verbal immediacy, discussed modes and semiotic resources, or sign systems, which indicated resources of meaning making, such as language, visual images, and music. They also mentioned that mode refers to “the channel (e.g., auditory, visual or tactile, for example) through which semiotic activity takes place” (O’Halloran, 2005, p. 20). The term “multimodality” also refers to a set of semiotic resources that presents and represents communication using diverse communicational modes like the image, gesture, gaze, posture (Jewitt, 2011) as well as digital sources (Erfanian Mohammadi et al., 2019). O’Halloran (2008, 2011) revealed that human communication is basically multimodal with different semiotic resources contributing to the meaning making of the classroom teaching and learning. O’Halloran (2011) also conceptualized multimodal discourse analysis (MDA) as relatively new domain that invites various theoretical perspectives. MDA refers to “theory and analysis of semiotic resources and the semantic expansions that occur as semiotic choice combine in multimodal phenomena” (p. 121). Velez and Cano (2012) mentioned that teachers’ use of non-verbal immediacy or semiotic resources can bridge the gap in interaction with learners as this behavior can strengthen closeness and non-verbal communication between interlocutors. It is the duty of teachers to convey emotional states, friendliness, closeness, sense of belonging (Zhang and Sapp, 2008). Numerous investigations have verified the concept that non-verbal immediacy augmented fondness in teacher-learner relationships educational contexts.

Verbal and non-verbal immediacy derives from interpersonal attraction theory (Mehrabian, 1969). In educational contexts, Byrne et al. (1973) defined interpersonal attraction theory as teachers’ emotional evaluation of learners. Allen et al. (2006) stated that people have (non-orally, orally, or emblematically) tendencies toward those individuals who adore. Based on interpersonal interaction theory, reinforcement is regarded as an important concept. Based on this principle, people are attracted to satisfying relationships. Consequently, teachers’ immediacy conducts in interaction with learners are regarded as rewarding which boost the extent of collaboration and commitment of learners in the educational contexts (Allen et al., 2006). Moreover, Aristotle’s theory of rhetoric can explicate the concept of teacher immediacy. Nayernia et al. (2020) stated that based on this theory, the word “convincing” can be categorized into personality, awakening emotion, and logos, which can influence the audience. Bizzell and Herzberg (2001) mentioned that individuals should have a good personality to create trustworthiness.

Teachers’ immediacy is associated with loving pedagogy that is a quite popular concept in SLA. Yin et al. (2019) asserted that teaching is not limited to the transfer of knowledge and should be beyond academic outcomes. They stated that effective teaching occurs in a loving environment to increase learners’ emotional status, classroom interaction, motivation, social ability, personality, and mental health. They also believed that education and instruction stem from pedagogical love. They maintained that educators can employ loving pedagogy in their instruction to instruct learners through love. In this respect, Loreman (2011) proposed a theoretical model of pedagogical love by considering the religious, emotional, and logical dimensions of human life. He incorporated some positive features, including kindness, passion, empathy, bonding, intimacy, sacrifice, forgiveness, and community, and acceptance into pedagogical love. He mentioned that love can bring out positive educational experiences. Barcelos and Coelho (2016) also stated that pedagogy of love is an integration of some positive psychological constructs, such as ethics, growth, care, respect, freedom, and dialogue. Moreover, they maintained that approving learners’ emotional states and aptitudes and considering language and love as two critical components of language learners’ academic development will contribute educators to consider the efficiency of love in their methodologies and instructional techniques. Barcelos (2020) stated that practicing pedagogical love can have some positive results for EFL learners and educators including their enhanced motivation, activity, self-sufficiency, social communication skills, creativity, resilience, academic performance, academic engagement, pedagogical success, and etc. Barcelos and Coelho (2016) also indicated to the limitation of research on use of pedagogical love in language education since it is a new topic for SLA practitioners. In the field of second language acquisition (SLA), Wang et al. (2022) highlighted the conceptualization and application of loving pedagogy in SLA research and practice. They proposed a practical model of loving pedagogy for SLA practitioners.

Some studies have been done on the relationship between teacher immediacy and learners’ positive and negative emotions. Regarding negative emotions, Tonsing (2018) found a negative correlation between teacher immediacy and learner anxiety. He argued that learners, who recognize teacher immediacy like telling their sources of apprehension, can significantly diminish their foreign language anxiety. Ballester (2015) also investigated the relationship between anxiety and teacher immediacy. He argued that lack of self-confidence leads to the lack of immediacy among teachers, and this type of deficiency results in learner anxiety. He mentioned that foreign language education unavoidably necessitates a comfortable atmosphere, where learners feel relaxed enough to contribute and cooperate with peers and teachers. Mazer et al. (2014) investigated the way learners’ negative emotions are affected by teacher non-verbal immediacy. Their study revealed that teacher non-verbal immediacy behaviors were significantly correlated with learners’ irritation, nervousness, embarrassment, desperateness, and boredom.

On the other hand, some studies have verified the positive relationship between teacher non-verbal and verbal immediacy and learners’ positive emotional constructs. Liu (2021) found a positive relationship between learner motivation and teacher immediacy. He used Keller’s (1987) ARCS model in order to explain this correlation. He argued that the arousal of motivation needs four features of ARCS model including “attention,” “relevance,” “confidence,” and “satisfaction.” Getting learners’ attention as one of the significant features to motivate learners for language learning teacher immediacy. He mentioned that learners will involve in language learning unless they do not pay attention to teachers. Wijaya (2017) also investigated the effect of teacher immediacy on learners’ motivation. His study showed that teachers’ immediacy significantly predicts learner motivation. He argued that educators are inclined to foster their learners’ level of motivation by reinforcing their communication with them. Seifu and Gebru (2012) argued that both non-verbal and verbal immediacy behaviors were significantly correlated with learners’ internal motivation in educational contexts.

Regarding the relationship between teacher immediacy and academic engagement, Derakhshan (2021) pointed out that learners’ engagement can be significantly improved by their instructors’ trustworthiness and non-verbal immediacy. He justified his findings by interpersonal attraction theory. He argued that educators can support their learners by using gestures, employing back-channels, and staring at learners during instruction. Dixson et al. (2017) also investigated the effect of online immediacy types on learner engagement. They found that and use of medium and image are significantly correlated with learner engagement. Pishghadam et al. (2021), in their study, revealed that teacher’s immediacy behaviors, language proficiency and credibility were significantly correlated with learners’ academic engagement. Xie and Derakhshan (2021) stated that teachers’ close affinity or immediacy influences professional commitment along with learners’ academic performance and enjoyment in educational contexts. In the following, the positive emotional construct, called enjoyment, and its relationship with verbal and non-verbal immediacy will be discussed.

### Foreign Language Enjoyment

According to Csikszentmihalyi (2014), foreign language enjoyment refers to “good emotional states coming from breaking through homeostatic limits and stretching beyond oneself to accomplish something new or even unexpected, especially in the face of some difficult tasks” (p. 201). Dewaele and MacIntyre (2016) also defined it as “a complex emotion, capturing interacting dimensions of challenge and perceived ability” (p. 216). Boudreau et al. (2018) also pointed out that this construct is described as a multifaceted dynamic emotional state, and it happens during the performance or accomplishment in an activity. Therefore, according to Csikszentmihalyi (2008) enjoyment is regarded as “a sense of novelty and of accomplishment” (p. 46). Mierzwa (2019) stated that learners’ feelings of being successful in instructive tasks along with making out the subject matters, are the features of enjoyment. Dewaele and Alfawzan (2018) argued that the construct of enjoyment includes facets, including intelligent focus, sharp attention, and ideal challenge, and it is an influential stimulus to foreign language learning. Hagenauer and Hascher (2014) also categorized the construct of enjoyment into behavioral, mental, emotional, expressional, and psychological dimensions. Han and Wang (2021) stated that the psychological component highlights emotions, and mainly, feeling of fulfillment, and desire felt throughout the educational procedure. Moreover, they mentioned that the mental dimension of enjoyment is related to the positive evaluation of the situation. Furthermore, they maintained that the motivational dimension is associated with learners’ capability to feel good by sensitively and physically inspiring them, to do their best in upcoming foreign language activities. Pekrun and Linnenbrink-Garcia (2014) stated that the enjoyment construct of foreign language learners derives from two theories, including Fredrickson’s (2001) broaden-and-build theory of positive emotions and Pekrun’s (2006) control-value theory of achievement emotions. MacIntyre and Gregersen (2012) used Fredrickson’s (2001) broaden-and-build theory to explicate the effect of enjoyment in foreign language learning, and they contended that enjoyment expedites the growth of learners’ thought-action repertoire to be proficient in language learning, and contribute them to increase language knowledge. Furthermore, they argued that control-value theory presumes that positive accomplishment feelings can inspire learners to endeavor, to be more imaginative and malleable in coping with troubles, and to control themselves appropriately. Balaž et al. (2021) also employed Pekrun’s (2006) control-value theory to justify the role of foreign language enjoyment in L2 learning achievement. They argued that enjoyment significantly stimulates activity-focused emotion, which, in turn, significantly influences learners’ language proficiency.

Some investigations have been done on the origins of foreign language enjoyment among learners. Dewaele et al. (2018b) found that foreign language learners’ enjoyment is significantly correlated with positive attitudes toward the foreign language, including the foreign language instructor, teachers’ talk in the educational context, and the time spent by learners on communicating with teacher and peers. Li et al. (2018) found that Chinese EFL learners have internal and external causes of foreign language enjoyment. They argued that the feeling of being successful, particularly in demanding activities, novelty experience, and satisfaction in obviating the difficulties are the primary internal causes of foreign language enjoyment. They also stated that encouraging classroom environment, teacher support, peer engagement, and cooperation in doing activities are the main external causes of foreign language enjoyment. Jiang and Dewaele (2019), in their study, revealed that teacher-related variables influence Chinese EFL learners’ enjoyment and learner-internal variables affect learners’ foreign language classroom anxiety. Dewaele et al. (2019) also revealed that foreign language enjoyment is significantly affected by teacher-related variables. These investigations accentuated the context-dependent effect of internal and external sources on the foreign language learner’s enjoyment (Joe et al., 2017).

Foreign language enjoyment has been considered as one of the constructs of positive emotional states in positive psychology (Wang et al., 2021). Traditionally, psychologists were concerned primarily with the students’ and educators’ negative emotional states, and they tried to diminish them (Derakhshan et al., 2021). Some studies, in the realm of foreign language learning, have undergone a positive rebirth, mostly the change of concentration from investigating negative emotions, particularly foreign language anxiety, to positive emotions (MacIntyre et al., 2020; Guo, 2021). In recent years, positive psychology, as a modern approach in educational contexts, attempted to enlighten the favorable educational circumstances and contexts for the accomplishment of students and educators (Jiang, 2020). Therefore, the attention shifted from negative emotions to positive emotional states like enjoyment, academic engagement, resilience, grit, well-being, emotional regulation, and positive teacher-learner relationship to improve their possible outcomes in the workplace (Buric and Macuka, 2018). Earlier investigations have mainly focused on the negative emotions of language teachers in numerous cultural contexts, because of the challenging nature of instruction (King and Ng, 2018). However, some studies have found a significant correlation between foreign language enjoyment and grit (Teimouri et al., 2020; Derakhshan, 2021; Yang, 2021), wellbeing, and resilience (Ergün and Dewaele, 2021), and academic engagement (Zeng, 2021).

In order to highlight the significant effect of positive constructs of psychology in foreign language instruction, and to emphasize the inspection of educators’ positive emotions and experiences, this review ponders into the relationship between foreign language enjoyment and verbal and non-verbal immediacy, as two positive psychological constructs, in pedagogical contexts.

### The Role of Teacher Verbal and Non-verbal Immediacy in Learner Foreign Language Enjoyment

Few studies have been done on the relationship between teacher immediacy and learners’ foreign language enjoyment. Wang (2021) pointed out that teachers are responsible for establishing rapport with learners, and he stated that teachers’ friendship with learners is a firm result of learner’ emotional growth, including enjoyment, resilience, grit, and hope. Dewaele et al. (2018a) also stated that “good language teacher needs to be in a position to manage the emotional tenor of the classroom. This means not only should they be able to harness the emotions of their learners, but also be able to regulate their own emotions to ensure they are in the right frame of mind to create positive rapport with learners, generate enjoyment and manage any anxieties” (p. 126). Dewaele et al.’s (2019) study revealed that immediacy and foreign accent as teachers’ features significantly predicted learner foreign language enjoyment. They argued that teachers’ performance in raising learners’ enjoyment is better than decreasing their anxiety. Therefore, they stated that teacher immediacy and encouragement to use foreign language motivates learners to progress. They added that mentioned that teachers’ behaviors such as immediacy, stroke, and respect toward learners have significantly influenced learner enjoyment. They maintained that instructors should try to build up enjoyment by having a positive relationship with learners.

Pavelescu and Petrić (2018) indicated that teacher immediacy has the capability to reinforce learners’ emotions. They asserted that a positive learner-teacher relationship can produce operational managing mechanisms, and inspire learners to enjoy the contexts. Mercer and Dörnyei (2020) suggested that the committees for teacher employment should consider teacher support and immediacy in the employment as one of useful ways to increase foreign language learner enjoyment in the classroom. They should ask teachers to employ strategies to create friendship in order to enhance foreign language learner enjoyment and engagement. Hsu (2005), in another study, found a significant correlation between learner motivation and teacher immediacy. He argued that learner motivation can be theorized as learners’ energy and determination to perform efficiently and attain to their prospective in educational contexts. He considered motivation as a main factor in learners’ foreign language enjoyment. In line with Hsu (2005) and Rocca (2007) highlighted the importance of immediacy behaviors in learners’ emotions. He also justified his results by expressing that teacher immediacy can stimulate learners to work harder and enjoy in educational contexts.

Titsworth et al. (2010), in their study, investigated the role of teacher non-verbal immediacy on learners’ enjoyment, pride, and hope. Their study showed that teacher immediacy reduced the extent of learners’ emotional distresses, and it significantly predicted learner pride, hope, and joy. They mentioned that teacher non-verbal immediacy “decreased the amount students felt they had to hide their emotional responses in class, increasing their pride, hope, and enjoyment.” Titsworth et al. (2013) verified the influence of teacher behavior, such as teacher immediacy and clarity, on learner emotions, such as pride, enjoyment, and hope. They mentioned that positive emotions are triggered learners “feel in control of activities and outcomes that are subjectively important” (p. 15). When learners realize emotional support from teachers, and they do not require to control their feelings via numerous methods of emotional work, their sense of control probably develops and this arouses the likelihood of positive emotions (Titsworth et al., 2013). Holzberger et al. (2019) found out that teacher immediacy and affinity developed learner enjoyment. They argued that learner emotional support can significantly predict leaners’ emotional developments. Lei et al. (2018) found out that teacher support and non-verbal immediacy significantly predicted positive emotions such as enjoyment, interest, hope, pride, and relief; while they had a negative correlation with anxiety as a negative emotion. Their study also revealed that the relationship between teacher immediacy and learners’ positive emotions was stronger than western European and American learners. However, this correlation was weak, but strong, for East Asian learners.

Bayat, Shirvan, and Barabadi, in their study, investigated teachers’ non-verbal immediacy as multimodal corrective feedback and learners’ enjoyment. They operationalized multimodality in non-verbal immediacy as body postures and movements such as gesture, gaze, facial expressions or the use of technological tools which accompany teachers’ corrective feedback. They analyzed multimodal corrective feedback by systemic functional multimodal discourse analysis. Their study revealed that teacher’s multimodality in their corrective feedback extended the central aspects of enjoyment by increasing the learners’ responsiveness to their errors, amplifying their concentration on the correct form, and intensifying the prominence of their corrective feedback. Using systemic functional multimodal discourse analysis, Yunita et al. (2022) indicated that building student-teacher relationships through some semiotics like clapping and thumbs up can bring enjoyment to younger language learners.

## Conclusion and Implications

This review investigated the role of teacher verbal and non-verbal immediacy in learners’ foreign language enjoyment in class. This review improves the instructive knowledge of scholars who are interested in learners’ and teachers’ emotions. Concerning the related literature about the positive role of learner-teacher rapport, in particular, teacher immediacy, in learner enjoyment, it is worth noting that learners should be helped to control and regularize their feelings in instructive contexts. Learners can regulate their feelings to increase their enjoyment, and this issue can stimulate instructors to take into account learners’ positive emotions in classroom contexts. The growth of immediacy among teachers and enjoyment among learners to involve actively in classrooms, can be regarded as one of the pedagogical implications of this review. Learners can employ opportunities that instructors provide to express their thoughts and emotions, which can decrease their stress in front of other learners and inspire them to be responsible for their learning. This study can also encourage teachers, teacher educators, and policymakers to consider EFL learners’ behaviors and their foreign language enjoyment. Also, being aware of learners’ personality traits may encourage teachers to do their best to bring passion to EFL learning contexts. Therefore, L2 instructors are required to talk to learners about their internal and external motivation and ask about their problems to improve learners’ points of view, motivation, and enjoyment to engage in educational contexts. They can increase other positive emotions such as pride, grit, and hope, and reduce negative feelings such as communication apprehension, disengagement, etc., in their classes. Moreover, teacher immediacy and considering learners’ requirements should be one of the rudiments of teaching in language education environments to achieve instructional outcomes. To do so, teachers need to reconstruct and modify the instructional materials. They can find authentic and interactive materials to increase learners’ foreign language enjoyment to attain better results. Incorporating technology into classroom contexts can be a pleasant way to increase learners’ foreign language enjoyment. Using games in the classroom can also arouse learner enjoyment. This can diminish learners’ cognitive load and despair, and arouse their enthusiasm and their concentration on language learning contexts. Teacher immediacy may persuade teachers to change their approach to teaching by incorporating positive emotions in their methodology to increase learners’ foreign language enjoyment. Consequently, they can provide warming-up tasks for learning contexts and brainstorm with learners to increase learner foreign language enjoyment. The challenging projects, lectures, conferences, and workshops may reduce learners’ foreign language enjoyment in academic contexts and put additional strain on learners. In order to motivate and increase learner enjoyment, providing learners with easy-to-difficult questions can be helpful, and teachers can change their assessment approach in classrooms. Teachers can manage the time of classrooms regularly to provide time for learners to communicate and enjoy their learning. Providing a competitive educational context through quizzes boosts learner engagement and enjoyment. Unplanned quizzes are primarily significant to inspire less engaged, and unprepared learners. Collaboration is another way for teachers to increase learners’ foreign language enjoyment. Allowing learners to cooperate can make them keep information for a long time. Cooperation also makes learners enhance critical thinking which makes the instruction more enjoyable. Teachers should accept learners’ creativity. They can provide learners the freedom to make projects and assess their peers’ assignments to increase learner enjoyment.

Besides, teacher educators can exploit the related studies through consideration of the instructors’ immediacy and their rapport with the learners. They can identify teacher behavioral characteristics that contribute to increasing students’ level of foreign language enjoyment and take appropriate actions to increase replicating such behaviors. They can hold workshops and provide some strategies to improve teacher immediacy. They can also emphasize modeling immediacy, taking action and improving listening, and trying not to interrupt learners while they are speaking. According to Gourneau (2005, p. 1), “effective attitudes and actions employed by teachers can ultimately make positive differences in the lives of their students.” Therefore, basically, as an example of positive teacher communication-relational behavior, teacher immediacy, can be clearly instructed to pre- or in-service EFL teachers as inspiring behaviors to enhance instruction. These behaviors include building a good rapport, paying attention to learners, making eye contact, promoting in-class discussion, praising student achievements, having a sense of humor, smiling, calling out names, and nodding. They can give some instructions to use gestures in communication with learners. Moreover, some recommendations, such as using non-monotonous speech, smiling during the speech, looking to the whole class during talking, and having a relaxed posture can be presented in the workshops. Teacher educators can assess and validate the effectiveness of instructors’ immediacy on learners’ engagement and enjoyment in EFL contexts. They should develop confidence and competence among in-service teachers to entice learners’ interests and engage them in the learning process.

### These Stakeholders

Policymakers can develop programs that help learners enjoy when learning a language and amplify their academic engagement. They can positively support learners and make a context in which learners can take part in positive behaviors. They can hold academic workshops to help teachers control immediacy behaviors. They can provide interesting facilities, technology, and positive learning contexts for increasing positive behaviors among learners. The importance of enjoyment and immediacy may make consultants expand their agendas to diagnose learners’ reasons for unhappiness, and the obstacles they cope with within language learning. They can also offer some suggestions to increase the efficiency of teacher immediacy.

## Suggestions for Further Research

Future studies may consist of investigating the influence of other teacher variables such as extroversion, and introversion on learners’ positive behaviors such as enjoyment, grit, pride, and engagement. Teachers’ negative emotions such as boredom, burnout, apprehension, and exhaustion and their role in learners’ foreign language enjoyment should be examined. Other studies can be done to investigate the effect of teacher stroke on learners’ academic engagement and enjoyment. Longitudinal studies are required to shed light on the effect of interpersonal emotions on language learning and learners’ positive emotions. Future studies can investigate the effects of teacher immediacy on learners’ working memory and long-term memory. Moreover, the effects of teacher immediacy on the improvement of language skills ought to be considered in detail. Further studies are needed to determine teacher immediacy in traditional and digital contexts to illuminate how these contexts may affect learners’ positive and negative emotions. Besides, further research can be done to investigate the gender effect and teacher proficiency level on teacher immediacy in language learning contexts. Finally, future studies should determine the relationship between EFL teachers’ immediacy and emotional intelligence in foreign language contexts.

## Ethics Statement

The studies involving human participants were reviewed and approved by the Zhejiang Gongshang University Academic Ethics Committee. The patients/participants provided their written informed consent to participate in this study.

## Author Contributions

All authors listed have made a substantial, direct, and intellectual contribution to the work, and approved it for publication.

## Conflict of Interest

The authors declare that the research was conducted in the absence of any commercial or financial relationships that could be construed as a potential conflict of interest.

## Publisher’s Note

All claims expressed in this article are solely those of the authors and do not necessarily represent those of their affiliated organizations, or those of the publisher, the editors and the reviewers. Any product that may be evaluated in this article, or claim that may be made by its manufacturer, is not guaranteed or endorsed by the publisher.

## References

[B1] Allen M. Witt P. L. Wheeless L. R. (2006). The role of teacher immediacy as a motivational factor in student learning: using meta-analysis to test a causal model. *Commun. Educ.* 55 21–31. 10.1080/03634520500343368

[B2] Andersen J. (1979). “Teacher immediacy as a predictor of teaching effectiveness,” in *Communication Yearbook*, Vol. 3 ed. Nimmo D. (New Brunswick, NJ: Transaction Books), 543–559. 10.1080/23808985.1979.11923782

[B3] Balaž B. Hanzec Marković I. Brajša-Žganec A. (2021). The exploration of the relationship between positive achievement emotions and academic success: testing the assumptions of the control-value theory of achievement emotions. *Educ. Dev. Psychol.* 38 77–87. 10.1080/20590776.2020.1856623

[B4] Ballester E. P. (2015). Verbal and nonverbal teacher immediacy and foreign language anxiety in an EFL university course. *Porta Linguarum* 23 9–24.

[B5] Barcelos A. M. F. (2020). “Revolutionary love and peace in the construction of an English teacher’s professional identity,” in *Peacebuilding in Language Education*, eds Oxford R. Olivero M. Harrison M. Gregersen T. (Bristol: Multilingual Matters), 96–109. 10.21832/9781788929806-011

[B6] Barcelos A. M. F. Coelho H. S. H. (2016). “Language learning and teaching: what’s love got to do with it?,” in *Positive psychology in SLA*, eds MacIntyre P. D. Gregersen T. Mercer S. (Derby: Multilingual Matters), 130–144. 10.21832/9781783095360-006

[B8] Bizzell P. Herzberg B. (2001). *The Rhetorical Tradition: Readings From Classical Times To The Present.* Boston, MA: Bedford.

[B9] Boudreau C. MacIntyre P. Dewaele J. M. (2018). Enjoyment and anxiety in second language communication: an idiodynamic approach. *Stud. Second Lang. Learn. Teach.* 8 149–170. 10.14746/ssllt.2018.8.1.7

[B10] Buric I. Macuka I. (2018). Self-efficacy, emotions and work engagement among teachers: a two wave cross-lagged analysis. *J. Happiness Stud.* 19 1917–1933. 10.1007/s10902-017-9903-9

[B11] Byrne D. Clore G. L. Griffitt W. Lamberth J. Mitchell H. E. (1973). When research paradigms converge: confrontation or integration? *J. Pers. Soc. Psychol.* 28 313–320. 10.1037/h0035105

[B12] Csikszentmihalyi M. (2008). *Flow: The Psychology Of Optimal Experience.* New York, NY: Harper Perennial.

[B13] Csikszentmihalyi M. (2014). *Flow And The Foundations Of Positive Psychology.* Dordrecht: Springer. 10.1007/978-94-017-9088-8

[B14] Derakhshan A. Kruk M. Mehdizadeh M. Pawlak M. (2021). Boredom in online classes in the Iranian EFL context: sources and solutions. *System* 101:102556. 10.1016/j.system.2021.102556

[B15] Derakhshan A. (2021). The predictability of turkman students’ academic engagement through persian language teachers’ nonverbal immediacy and credibility. *J. Teach. Persian Speakers Other Lang.* 10 3–26.

[B16] Dewaele J. M. Alfawzan M. (2018). Does the effect of enjoyment outweigh that of anxiety in foreign language performance? *Stud. Second Lang. Learn. Teach.* 8 21–45. 10.14746/ssllt.2018.8.1.2

[B19] Dewaele J. M. Witney J. Saito K. Dewaele L. (2018b). Foreign language enjoyment and anxiety: the effect of teacher and learner variables. *Lang. Teach. Res.* 22 676–697. 10.1177/1362168817692161

[B18] Dewaele J. M. Gkonou C. Mercer S. (2018a). “Do ESL/EFL teachers’ emotional intelligence, teaching experience, proficiency and gender affect their classroom practice?,” in *Emotions In Second Language Teaching*, ed. Agudo J. D. M. (Cham: Springer), 125–141. 10.1007/978-3-319-75438-3_8

[B20] Dewaele J. M. Magdalena A. F. Saito K. (2019). The effect of perception of teacher characteristics on Spanish EFL learners’ anxiety and enjoyment. *Modern Lang. J.* 103 412–427. 10.1111/modl.12555

[B21] Dewaele J.-M. MacIntyre P. D. (2016). “Foreign language enjoyment and foreign language classroom anxiety: the right and left feet of FL learning?,” in *Positive Psychology in SLA*, eds MacIntyre P. D. Gregersen T. Mercer S. (Bristol: Multilingual Matters), 215–236. 10.21832/9781783095360-010

[B22] Dixson M. D. Greenwell M. R. Rogers-Stacy C. Weister T. Lauer S. (2017). Nonverbal immediacy behaviors and online student engagement: Bringing past instructional research into the present virtual classroom. *Commun. Educ.* 66 37–53. 10.1080/03634523.2016.1209222

[B23] Erfanian Mohammadi J. Elahi Shirvan M. Akbari O. (2019). Systemic functional multimodal discourse analysis of teaching students developing classroom materials. *Teach. High. Educ.* 24 964–986. 10.1080/13562517.2018.1527763

[B24] Ergün P. Dewaele J.-M. (2021). Do well-being and resilience predict the foreign language teaching enjoyment of teachers of Italian? *System* 99 1–21. 10.1016/j.system.2021.102506

[B25] Fredrickson B. L. (2001). The role of positive emotions in positive psychology: the broaden-and-build theory of positive emotions. *Am. Psychol.* 56 218–226. 10.1037/0003-066X.56.3.218 11315248 PMC3122271

[B26] Frymier A. B. Goldman Z. W. Claus C. J. (2019). Why nonverbal immediacy matters: a motivation explanation. *Commun. Q.* 1 1–14. 10.1080/01463373.2019.1668442

[B27] Gourneau B. (2005). Five attitudes of effective teachers: implications for teacher training. *Essays Educ.* 13 1–8. 10.17761/2019-00015 30430916

[B28] Guo Y. (2021). Exploring the dynamic interplay between foreign language enjoyment and learner engagement with regard to EFL achievement and absenteeism: a sequential mixed methods study. *Front. Psychol.* 12:766058. 10.3389/fpsyg.2021.766058 34721246 PMC8554116

[B29] Hagenauer G. Hascher T. (2014). Early adolescents’ enjoyment experienced in learning situations at school and its relation to student achievement. *J. Educ. Train. Stud.* 2 20–30. 10.11114/jets.v2i2.254

[B30] Han Y. Wang Y. (2021). Investigating the correlation among Chinese EFL teachers’ self-efficacy, reflection, and work engagement. *Front. Psychol.* 12:763234. 10.3389/fpsyg.2021.763234 34803845 PMC8603392

[B31] Holzberger D. Praetorius A. K. Seidel T. Kunter M. (2019). Identifying effective teachers: the relation between teaching profiles and students’ development in achievement and enjoyment. *Eur. J. Psychol. Educ.* 34 801–823. 10.1007/s10212-018-00410-8

[B32] Hsu S. Y. (2005). Building language-learning environments to help technological university students develop English independent learning. *Jalt Call J.* 1 51–66. 10.29140/jaltcall.v1n2.r10

[B33] Inan-Kaya G. Rubie-Davies C. M. (2021). Teacher classroom interactions and behaviors: indications of bias. *Learn. Instr.* 78:101516. 10.1016/j.learninstruc.2021.101516

[B34] Jewitt C. (2011). *The Routledge Handbook of Multimodal Analysis*. London and New York, NY: Routledge.

[B35] Jiang Y. (2020). An investigation of the effect of teacher on Chinese university students’ foreign language enjoyment. *Foreign Lang. World* 196 60–68.

[B36] Jiang Y. Dewaele J. M. (2019). How unique is the foreign language classroom enjoyment and anxiety of Chinese EFL learners? *System* 82 13–25. 10.1016/j.system.2019.02.017

[B37] Joe H. K. Hiver P. Al-Hoorie A. H. (2017). Classroom social climate, self-determined motivation, willingness to communicate, and achievement: a study of structural relationships in instructed second language settings. *Learn. Individ. Dif.* 53 133–144. 10.1016/j.lindif.2016.11.005

[B38] Keller J. M. (1987). Development and use of the ARCS model of instructional design. *J. Instr. Dev.* 10 2–10. 10.1007/BF02905780

[B39] King J. Ng K. Y. S. (2018). Teacher emotions and the emotional labor of second language teaching. *Lang. Teach. Psychol.* 2 141–157. 10.21832/9781783099467-013

[B41] Lei H. Cui Y. Chiu M. M. (2018). The relationship between teacher support and students’ academic emotions: a meta-analysis. *Front. Psychol.* 8:2288. 10.3389/fpsyg.2017.02288 29403405 PMC5786576

[B42] Li C. Jiang G. Dewaele J. M. (2018). Understanding Chinese high school students’ foreign language enjoyment: validation of the Chinese version of the foreign language enjoyment scale. *System* 76 183–196. 10.1016/j.system.2018.06.004

[B43] Liando N. V. F. (2010). Students vs. teachers’ perspectives on best teacher characteristics in EFL classrooms. *TEFLIN J.* 21 118–136.

[B44] Liu B. (2016). Effect of L2 exposure: from a perspective of discourse markers. *Appl. Linguist. Rev.* 7 73–98. 10.1515/applirev-2016-0004

[B45] Liu W. (2021). Does teacher immediacy affect students? A systematic review of the association between teacher verbal and non-verbal immediacy and student motivation. *Front. Psychol.* 12:713978. 10.3389/fpsyg.2021.713978 PMC8267458 34248809

[B46] Loreman T. (2011). *Love As Pedagogy.* Rotterdam: Sense. 10.1007/978-94-6091-484-3

[B47] MacIntyre P. D. Gregersen T. Mercer S. (2020). Language teachers’ coping strategies during the Covid-19 conversion to online teaching: correlations with stress, wellbeing and negative emotions. *System* 94:102352. 10.1016/j.system.2020.102352

[B48] MacIntyre P. Gregersen T. (2012). Emotions that facilitate language learning: the positive-broadening power of the imagination. *Stud. Second Lang. Learn. Teach.* 2 193–213. 10.14746/ssllt.2012.2.2.4

[B49] Malik S. K. (2012). Prospective teachers’ awareness about interpersonal skills: a comparative study. *Interdiscip. J. Contemp. Res. Bus.* 3 514–522. 10.1186/s12913-016-1423-5 27409075 PMC4943498

[B50] Mazer J. P. McKenna-Buchanan T. P. Quinlan M. M. Titsworth S. (2014). The dark side of emotion in the classroom: emotional processes as mediators of teacher communication behaviors and student negative emotions. *Commun. Educ.* 63 149–168. 10.1080/03634523.2014.904047

[B51] Mehrabian A. (1969). Some referents and measures of nonverbal behavior. *Behav. Res. Methods Instrum.* 1 203–207. 10.3758/BF03208096

[B52] Mercer S. Dörnyei Z. (2020). *Engaging Language Learners In Contemporary Classrooms.* Cambridge: Cambridge University Press.

[B53] Mierzwa E. (2019). Foreign language learning and teaching enjoyment: teachers’ perspectives. *J. Educ. Cult. Soc.* 10 170–188. 10.15503/jecs20192.170.188

[B54] Nayernia A. Taghizadeh M. Farsani M. A. (2020). EFL teachers’ credibility, nonverbal immediacy, and perceived success: a structural equation modelling approach. *Cogent Educ.* 7 1–15. 10.1080/2331186X.2020.1774099

[B55] O’Halloran K. (2008). Systemic functional-multimodal discourse analysis (SF-MDA): constructing ideational meaning using language and visual imagery. *Vis. Commun.* 7 443–475. 10.1177/1470357208096210

[B56] O’Halloran K. L. (2005). *Mathematical Discourse: Language, Symbolism And Visual Images.* London: Continuum.

[B57] O’Halloran K. L. (2011). “Multimodal discourse analysis,” in *Companion To Discourse*, eds Hyland K. Paltridge B. (London: Continuum), 120–137. 10.1080/13562517.2018.1527763

[B58] Pavelescu L. M. Petrić B. (2018). Love and enjoyment in context: four case studies of adolescent EFL learners. *Stud. Sec. Lang. Lear. Teach.* 8:73101. 10.14746/ssllt.2018.8.1.4

[B59] Pekrun R. (2006). The control-value theory of achievement emotions: assumptions, corollaries, and implications for educational research and practice. *Educ. Psychol. Rev.* 18 315–341. 10.1007/s10648-006-9029-9

[B60] Pekrun R. Linnenbrink-Garcia L. (2014). *International Handbook Of Emotions In Education.* New York, NY: Routledge. 10.4324/9780203148211

[B61] Peng J. E. (2020). Teacher interaction strategies and situated willingness to communicate. *Engl. Lang. Teach.* 74 307–317. 10.1093/elt/ccaa012

[B62] Peng J.-E. Zhang L. Chen Y. (2016). The mediation of multimodal affordances on willingness to communicate in the english as a foreign language classroom. *TESOL Q.* 51 302–331. 10.1002/tesq.298

[B63] Pishghadam R. Derakhshan A. Zhaleh K. Al-Obaydi L. H. (2021). Students’ willingness to attend EFL classes with respect to teachers’ credibility, stroke, and success: a cross-cultural study of Iranian and Iraqi students’ perceptions. *Curr. Psychol.* 3, 1–15. 10.1007/s12144-021-01738-z

[B64] Pladevall-Ballester E. (2015). Exploring primary school CLIL perceptions in Catalonia: students’, teachers’ and parents’ opinions and expectations. *Int. J. Bilingual Educ.* 18 45–59. 10.1080/13670050.2013.874972

[B65] Reeve J. (2005). *Understanding Motivation And Emotion.* New York, NY: Wiley.

[B66] Rocca K. (2007). Immediacy in the classroom: research and practical implications. *Commun. Educ.* 50 283–297.

[B67] Seifu A. Gebru A. (2012). Relationship between teacher verbal and non-verbal immediacy and student motivation in EFL classes. *Ethiopian J. Educ.* 32 71–104.

[B68] Sheybani M. (2019). The relationship between EFL learners’ Willingness to Communicate (WTC) and their teacher immediacy attributes: a structural equation modelling. *Cogent Psychol*. 6, 1–14. 10.1080/23311908.2019.1607051

[B69] Teimouri Y. Plonsky L. Tabandeh F. (2020). L2 grit: passion and perseverance for second-language learning. *Lang. Teach. Res.* 107 1–18. 10.1177/1362168820921895

[B70] Titsworth S. McKenna T. P. Mazer J. P. Quinlan M. M. (2013). The bright side of emotion in the classroom: do teachers’ behaviors predict students’ enjoyment, hope, and pride? *Commun. Educ.* 62 191–209. 10.1080/03634523.2013.763997

[B71] Titsworth S. Quinlan M. M. Mazer J. P. (2010). Emotion in teaching and learning: development and validation of the classroom emotions scale. *Commun. Educ.* 59 431–452. 10.1080/03634521003746156

[B72] Tonsing K. N. (2018). Instructor immediacy and statistics anxiety in social work undergraduate students. *Soc. Work Educ.* 37 223–233.

[B73] Velez J. J. Cano J. (2012). Instructor verbal and nonverbal immediacy and the relationship with student self-efficacy and task value motivation. *J. Agric. Educ.* 53 87–98. 10.5032/jae.2012.02087

[B74] Wang X. (2021). Cognitive and affective learning in english as a foreign language/english as a second language instructional-learning contexts: does teacher immediacy matter? *Front. Psychol.* 12:7597848. 10.3389/fpsyg.2021.759784 34721239 PMC8548565

[B75] Wang Y. Derakhshan A. Pan Z. (2022). Positioning an agenda on a loving pedagogy in second language acquisition: conceptualization, practice, and research. *Front. Psychol.* 13, 1–7. 10.3389/fpsyg.2022.894190 35668974 PMC9164106

[B76] Wang Y. L. Derakhshan A. Zhang L. J. (2021). Researching and practicing positive psychology in second/foreign language learning and teaching: the past, current status and future directions. *Front. Psychol.* 12:731721. 10.3389/fpsyg.2021.731721 34489835 PMC8417049

[B77] Wen W. P. Clement R. (2003). A Chinese conceptualization of willingness to communicate in ESL. *Lang. Cult. Curriculum* 16 18–38. 10.1080/07908310308666654

[B78] Wijaya R. K. (2017). Students’ perception of teachers nonverbal immediacy behavior toward students’ attitude and motivation in learning English. *ELT Worldwide* 4 75–91. 10.26858/eltww.v4i1.3198

[B79] Witt P. L. Kerssen-Griep J. (2011). Instructional feedback I: the interaction of facework and immediacy on students’ perceptions of instructor credibility. *Commun. Educ.* 60 75–94. 10.1080/03634523.2010.507820

[B80] Xie F. Derakhshan A. (2021). A conceptual review of positive teacher interpersonal communication behaviors in the instructional context. *Front. Psychol.* 12: 708490. 10.3389/fpsyg.2021.708490 34335424 PMC8319622

[B81] Yang P. (2021). Exploring the relationship between Chinese EFL students’ grit, well-being, and classroom enjoyment. *Front. Psychol.* 12:762945. 10.3389/fpsyg.2021.762945 34777167 PMC8586070

[B82] Yin L. C. Loreman T. Majid R. A. Alias A. (2019). The dispositions towards loving pedagogy (DTLP) scale: instrument development and demographic analysis. *Teach. Teach. Educ.* 86 1–9. 10.1016/j.tate.2019.102884

[B83] Yunita W. Abdullah F. Mellan M. Hidayati A. N. Ardi H. (2022). Managing english young learners’ classroom activities through gestures: a multimodal perspective. *Jurnal Obsesi* 6 2962–2973. 10.31004/obsesi.v6i4.2007

[B84] Zeng Y. (2021). A review of foreign language enjoyment and engagement. *Front. Psychol.* 12:737613. 10.3389/fpsyg.2021.737613 34456835 PMC8397506

[B85] Zhang Q. Sapp D. A. (2008). A burning issue in teaching: the impact of perceived teacher burnout and nonverbal immediacy on student motivation and affective learning. *J. Commun. Stud.* 1 1–19.

